# Using routine health information data for research in low- and middle-income countries: a systematic review

**DOI:** 10.1186/s12913-020-05660-1

**Published:** 2020-08-25

**Authors:** Yuen W. Hung, Klesta Hoxha, Bridget R. Irwin, Michael R. Law, Karen A. Grépin

**Affiliations:** 1 University of Waterloo, School of Public Health and Health Systems, Waterloo, Canada; 2 Department of Health Sciences, Wilfrid Laurier University, Waterloo, Canada; 3 Centre for Health Services and Policy Research, The University of British Columbia, Vancouver, Canada; 4 School of Public Health, Hong Kong University, Pok Fu Lam, Hong Kong

**Keywords:** Routine health information systems, Low- and middle-income countries, Systematic review

## Abstract

**Background:**

Routine health information systems (RHISs) support resource allocation and management decisions at all levels of the health system, as well as strategy development and policy-making in many low- and middle-income countries (LMICs). Although RHIS data represent a rich source of information, such data are currently underused for research purposes, largely due to concerns over data quality. Given that substantial investments have been made in strengthening RHISs in LMICs in recent years, and that there is a growing demand for more real-time data from researchers, this systematic review builds upon the existing literature to summarize the extent to which RHIS data have been used in peer-reviewed research publications.

**Methods:**

Using terms ‘routine health information system’, ‘health information system’, or ‘health management information system’ and a list of LMICs, four electronic peer-review literature databases were searched from inception to February 202,019: PubMed, Scopus, EMBASE, and EconLit. Articles were assessed for inclusion based on pre-determined eligibility criteria and study characteristics were extracted from included articles using a piloted data extraction form.

**Results:**

We identified 132 studies that met our inclusion criteria, originating in 37 different countries. Overall, the majority of the studies identified were from Sub-Saharan Africa and were published within the last 5 years. Malaria and maternal health were the most commonly studied health conditions, although a number of other health conditions and health services were also explored.

**Conclusions:**

Our study identified an increasing use of RHIS data for research purposes, with many studies applying rigorous study designs and analytic methods to advance program evaluation, monitoring and assessing services, and epidemiological studies in LMICs. RHIS data represent an underused source of data and should be made more available and further embraced by the research community in LMIC health systems.

## Background

Routine health information systems (RHISs) collect and provide information at regular intervals on services and activities delivered in health facilities [1]. RHISs have been implemented in many low and middle-income country (LMIC) health systems to support resource allocation and day-to-day management decisions at facility, district, provincial, and national levels, as well as to facilitate strategy development and policy-making [2, 3]. Despite the fact that RHISs are being implemented at scale in many LMICs, and that they have been widely recognized as an important component of health systems strengthening [4, 5], prior studies have suggested that researchers continue to prefer using intermittent cross-sectional population-based surveys rather than RHISs data to conduct studies, including the monitoring of health programs and policy evaluations [6–8].

In order to improve health system performance, reliable, timely, and transparent data on health services are crucial [9, 10]. RHISs collect such data and thus could provide important insights into health system performance [4]. Substantial investments have been made in the development and strengthening of RHISs in many LMICs over the past two decades [5, 11], and interventions targeting data collection, processing, analysis, and dissemination have increased the accessibility of RHIS data [5, 12]. While early RHISs were established using paper-based health facility reports, newer web-based systems have been adopted in many LMICs over the last decade [13, 14]. The most common of these is the District Health Information System 2 (DHIS 2) platform, which is used as the foundation for the national health management information systems (HMIS) in at least 46 countries and has been piloted in at least another 21 countries [15]. Studies have shown that the implementation of newer information and communication technology systems, along with supportive feedback mechanisms to encourage their use in routine practice, can lead to substantial improvements in RHIS reporting and data quality [5, 13, 16, 17].

Despite the documented improvements in data quality, recent studies have shown a persistent underuse of RHIS data for research purposes in LMICs [8, 18]. A number of factors may contribute to the underuse of RHIS data. Numerous studies and commentators have questioned the usefulness of RHIS-sourced data to monitor and evaluate health services due to data quality concerns, such as incompleteness and inaccuracy [19–23]. Additionally, RHIS data are often not publicly available for secondary analyses, which further limits their use [24]. Due to these concerns, the research community has shown a persistent preference to use data sourced from intermittent cross-sectional population-based surveys rather than data sourced from RHISs to conduct research on health service utilization and policy evaluation in LMICs [8, 18, 25, 26]. However, population-based surveys also have drawbacks, including the fact that they may be costly [26] and are often unable to generate sufficient data at the district or other subnational-levels [27]. In addition, reliance on such data may encourage the use of potentially weak evaluation designs [8] and may make establishing an appropriate baseline challenging when trying to evaluate specific programs [28]. Intermittent cross-sectional population-based surveys themselves also suffer from a number of quality concerns and thus should not be considered the gold standard for estimating service coverage or other population-based estimates [29].

Given the potential of RHISs to play a greater role in the evaluation of health policy and programs and to monitor the performance of health systems, it is important to better understand the extent to which such data are currently being used in research studies. To date, there have been no systematic reviews of the use of RHIS data for research purposes beyond studies that were specific to malaria control [18], a gap this paper seeks to address. Specifically, we systematically reviewed the published literature to identify and describe the different ways in which RHIS data have been used in peer-reviewed research, including the types of health conditions studied. We also summarized the different methodologies that have been used to analyze RHIS data in research and the types of strategies that were applied to circumvent common RHIS data issues, such as incomplete or inaccurate data. It is our goal to provide guidance to other researchers who may be interested in using such data for research purposes by helping them to gain a better understanding on how such data have been successfully used in other contexts.

## Methods

This systematic literature review followed the Preferred Reporting Items for Systematic Reviews and Meta-Analyses (PRISMA) guidelines. Peer-reviewed published studies that used data from RHISs in LMICs were included in this study, where RHISs were defined as data systems designed to collect and generate information on services provided by health facilities at regular intervals of a year or less [1]. This included data systems that collect information on clinical service delivery, pharmaceuticals, or diagnostic service provision, as well as routine administrative management. Conversely, systems that collect individual-level data for clinical decision-making purposes and pilot systems to test the implementation of a new data collection component or method were not considered to be RHISs.

### Search strategy

Relevant studies were identified through an electronic search of four databases of peer-reviewed literature: PubMed, Scopus, EMBASE, and EconLit -- from inception through February 20, 2019, the date we launched the search. For each database, we identified studies that contained any of the following free text terms in their titles or abstracts: ‘routine health information system’, ‘health information system’, or ‘health management information system’, and any LMIC, as defined by the World Bank’s 2019 classifications (Appendix 1). Articles were included in the study if they met the following criteria: a) full-text article available in English, b) original research, and c) used data from a RHIS in at least one LMIC for research purposes. In order to be considered as having used data from a RHIS for research purposes, studies had to involve an analysis, either descriptive or analytical, of RHIS data, or applied RHIS data to inform their study design. We excluded studies that: a) only described RHISs, b) only described the administrative decision-making process, c) only focused on RHIS data collection issues, or d) only assessed RHIS data quality.

### Selection of studies

**Fig. 1:**
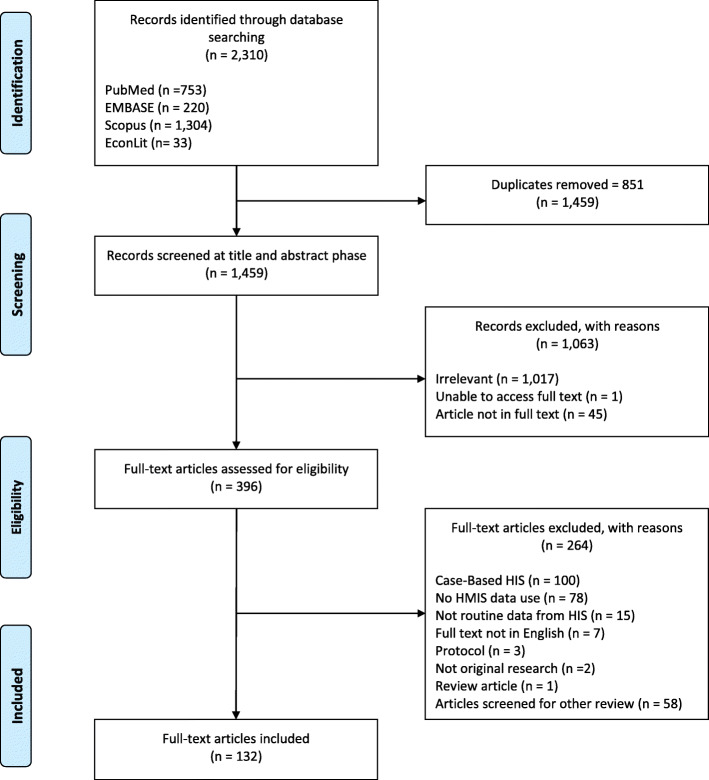
PRISMA flowchart of study identification and screening process of publications use RHIS data

Figure 1 shows the number of articles identified and retained at each stage of the systematic review process. After removing duplicates from the various database searches, we identified 1459 potential articles. Two reviewers independently screened the search results by title and abstract for inclusion eligibility. When there was insufficient information to determine eligibility at the title and abstract screening stage, the article was included for full-text screening. Full texts of the potentially eligible articles were then obtained and further screened for inclusion eligibility. At both stages, the reasons for excluding individual articles were recorded. The full-texts for all but one article were found. Disagreements at each stage were resolved through discussion. Where an agreement could not be reached, a third reviewer made the final determination.

### Data extraction and analysis

Two authors extracted data from all included studies using a piloted data extraction form. For each included article, data were extracted on study design, study objective, disease or health condition categories, study sample, description of RHIS data used, use of other data sources, analytic methods of RHIS data, strategies applied to circumvent data quality issues, and study findings. Due to the heterogeneity of the studies in terms of study design, study purpose, health conditions, and analysis methods, we thematically analyzed the studies according to research purpose, types of diseases studied, analytic methods applied, impact factor of journals in which the articles were published, and types of strategies used to circumvent RHIS data quality issues.

## Results

Of the 1459 unique articles retrieved from the database search, 132 studies met the inclusion criteria after full-text screening and were thus included in the review. The characteristics of these studies are presented in Table 1. Our review identified studies from 37 different countries. Three quarters of the studies were from Sub-Saharan African countries (74%), followed by South Asia (11%). The vast majority of the studies were published in the last decade, and more than half were published after 2014 (55%), suggesting an increase in the use of RHIS data for research purposes over time. Most of the studies included an analysis of RHIS data (97%), and a few used RHIS data to inform the study but did not describe analysis of RHIS data. One study, for example, used information from RHISs to justify for the selection of the indicators to be used at the individual-level in their study. Among the studies that analyzed RHIS data, most utilized an ecological study design (79%). Of those, more than half included statistical inferences (61%), while the remaining studies only used RHIS data for descriptive purposes (39%). Nearly a fifth of the studies were mixed methods or case studies (18%), a third of which included statistical analyses of RHIS data (33%). A quarter of articles included a description of how they managed missing data (25%), while only a small number of studies described how they detected and dealt with extreme values (14%).

**Table 1:** Characteristics of research studies that used RHIS data

		n	Percent
Geographical region			
East Asia and Pacific		8	6.1
Latin America and the Caribbean		9	6.8
Middle East and North Africa		2	1.5
South Asia		15	11.4
Sub-Saharan Africa		98	74.2
Year of publication			
< 2000		3	2.3
2000–2004		7	5.3
2005–2009		10	7.6
2010–2014		40	30.3
2015–2019		72	54.5
RHIS data as source or to inform study			
Data source		128	97.0
Inform study		4	3.0
Types of study design			
Ecological study - cross-sectional		13	9.8
Ecological study - longitudinal		51	38.6
Ecological study - descriptive		41	31.1
Case study		11	8.3
Mixed methods study		13	9.8
Cross-sectional study		1	0.8
Pre- and post-intervention study		1	0.8
Nested clustered randomized controlled trial		1	0.8
Data use purpose			
Program evaluation		67	50.8
Epidemiology		23	17.4
Monitoring and assessment of service provisions		30	22.7
Program description		6	4.5
Impact evaluation		4	3.0
Cost estimation		2	1.5
Health conditions/service type			
General (multiple aspects)		21	15.9
Secondary health utilization		2	1.5
General causes of death		1	0.8
Maternal and Child health/healthcare		12	9.1
Maternal health/healthcare		24	18.2
Child health/healthcare		11	8.3
Vaccine prevented childhood illnesses		10	7.6
Malaria		30	22.7
Malaria & HIV/AIDS		1	0.8
Malaria & other parasitic diseases		1	0.8
HIV and related diseases		8	6.1
Mental health/healthcare		3	2.3
Other diseases		5	3.8
Healthcare workforce and other resources		2	1.5
Data issue of RHIS: missingness			
Described how missing data was managed		33	25.0
No description of how missing data was managed		99	75.0
Data issue of RHIS: outlier			
Described how outlier was detected		19	14.4
No description of how outlier was detected		113	85.6

### Types of disease and research purpose

**Fig. 2:**
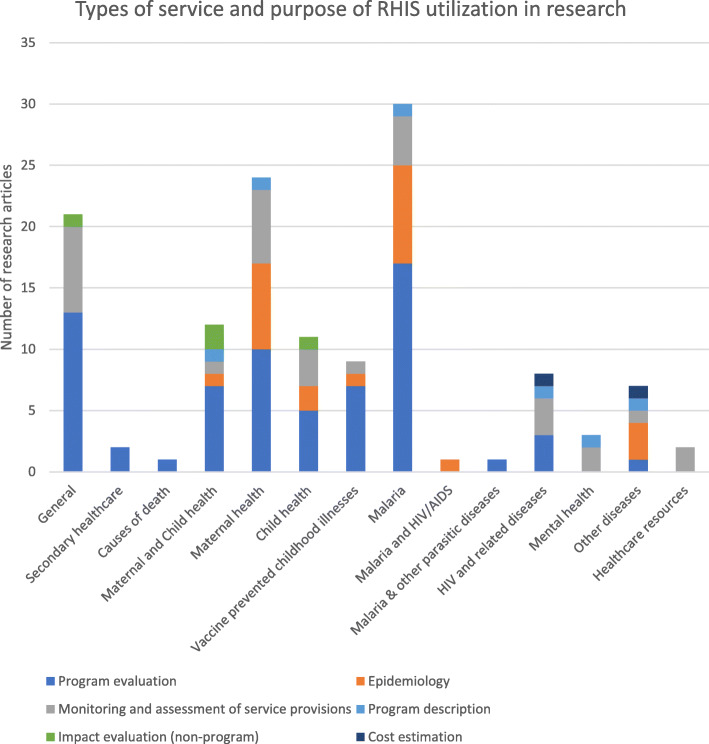
Types of service and research purpose of RHIS data use (*n* = 132)

Figure 2 shows the different research purposes for which RHIS data were used, along with the health topics investigated. The most common purpose of the studies was program evaluation (51%). RHIS data have been used to evaluate a wide range of interventions, ranging from programs that targeted specific diseases to interventions or policies that affected multiple types of diseases or health services. These included: the effect of malaria control strategies [30–36], user fee exemption policies [37–40], health financing schemes [41–44], interventions on health governance [45–53], the administration of new vaccines and vaccination campaigns [54–56], as well as community-level interventions such as approaches to enhance community participation and improve referrals from traditional birth attendants in increasing the demand for maternal and child care [57–59].

Additionally, RHIS data were used to monitor or assess service provision (23%) and to describe disease epidemiology (17%). Similar to the program evaluation studies, these studies also investigated a diverse set of health services and the allocation of healthcare resources. Some of these studies found large discrepancies between RHIS data and an estimated disease burden in populations or highlighted the lack of service provision. A few studies also used RHIS data to describe specific programs [60–64], conduct impact evaluations (non-programmatic) [65–68], and estimate costs [69, 70]. Most of the studies investigated a communicable disease (95%), of which malaria was most studied health condition (24%). A few studies focused on mental health (2%), diabetes mellitus (1%), and permanent tooth extraction (1%). Only two studies used RHIS data to research the health workforce or the equity of funding allocations [71, 72].

### Analytic methods using RHIS data

Among articles that conducted statistical analyses using RHIS data (*n* = 68), time series analyses to test or account for trends were most commonly performed (25%), followed by geostatistical analyses (16%), pre-post comparisons (15%), interrupted time series (ITS) (10%), and difference-in-difference analyses (7%). Other longitudinal analyses (13%), other cross-sectional analyses (12%), and scenario analysis on cost effectiveness (2%) were also conducted. Table 2 presents the range of methodologies identified across studies using RHIS data, as well as the corresponding articles.

**Table 2:** Types of analytic methods applied among studies that analyzed RHIS data

Data use purpose	Type of disease/service studied	Range of data (unit)	Level of aggregation	Analytic methods	Other information sources included	Reference
*Time series analysis*						
Epidemiology	Child health, malaria, tooth extraction	15 (year) - 120 (month)	Ward, municipal, district	Time series correlograms; ordinary least-squares regressions adjusted for seasonality and lag; non-linear time series correlation and regressions	GPS coordinates, Climate Hazards Group Infrared Precipitation with Station Data, satellite data, meteorological department data, program data	[73–76]
Program evaluation	General, maternal and child health, maternal health, vaccine prevented childhood illnesses, malaria	5 (year) - 168 (month)	Facility, district, region, nation	Ordinary least squares regression; negative binomial generalized linear model; random effects negative binomial regressions; switching regression methods weighted by propensity scores	Program data, program reports, data from Bureau of Statistics and Ministry of Health, Malaria Indicator Survey, Demographic Health Survey, Health Facility Survey, community survey, satellite data, sentinel site case-investigations/surveillance, abstraction from hospital registries	[33, 34, 37, 40, 54, 55, 58, 77–81]
Impact evaluation (non-program)	General	84 months (month)	Facility	Linear mixed-effect time-series analysis with a segmented regression parameterization	None	[82]
*Interrupted time series analysis*						
Program evaluation	General, maternal and child health, maternal health, malaria	53 (month) - 132 (month)	Facility, intervention vs. control groups, district	Generalized least square model with autoregressive structure; generalized least square model with controls, with autoregressive process and moving average process; segmented linear regression	Meteorology Department data, program data, facility survey	[38, 45, 83–86]
Impact evaluation (non-program)	Maternal and child health	44 (month)	District	Segmented linear regression with district fixed effect and clustered standard error at district level	Demographic Health Survey	[68]
*Difference-in-difference analysis*						
Program evaluation	General, child health, maternal health	4 (year) - 48 (month)	Facility, district, province	Ordinary least squares regression with and without propensity score matching; Wilcoxon rank-sum test on median difference-in-differences between facilities; descriptive comparison of means	Verified data from Performance-Based Financing system	[41, 42, 59, 87, 88]
*Pre-post comparison analysis*						
Program evaluation	Child health, maternal health, maternal and child health, vaccine prevented childhood illnesses, malaria, HIV or related diseases	2 (year) - 48 (month)	Facility, district	Chi-square test; Pearson correlation; Wilcoxon signed-rank test; paired sample t-test; linear regressions; Poisson regression; negative binomial regression; logistic regression	Bureau of Statistics data, program reports, Meteorological Department data, entomological sentinel surveys, Demographic and Health Survey, UN Interagency Group for Childhood Mortality Estimation(CME Info) database, abstraction from facility registers, community surveys, vital registry, provincial maternal death notification register	[35, 39, 48, 57, 89–93]
Impact evaluation (non-program)	Child health	26 (month)	District	Pearson chi-square test	District hospital registers, Safe and dignified burials for all deaths database	[67]
*Other longitudinal analysis*						
Epidemiology	Maternal health, malaria	12 (year) - 16 (year)	District	Chi-square test; negative binomial regression	Review of hospital death records	[94, 95]
Monitoring and assessment of service provision	HIV or related diseases	3 (year)	District	Descriptive comparison over time	Surveys with health facility managers	[96]
Program evaluation	Genera, child health, malaria, malaria and other parasitic diseases	3 (year) - 24 (month)	Facility, district, nation	Poisson regression to explore association between intervention coverage and disease burden; Mann–Whitney U Test to compare prevalence in intervention and non-intervention area; linear regression model; student t-test	Sentinel surveillance data, program reports, national facility and community survey, Bureau of Statistics data, program data	[47, 52, 66, 97–99]
*Geostatistical analysis*						
Epidemiology	Child health, malaria, malaria and HIV/AIDS, meningococcal meningitis	1 (year) - 520 (week)	District	Cluster analysis; cross-correlations of different spatial scales between time series of cases; Bayesian hierarchical Poisson model and smoothed model estimates plotted on district maps	Malaria Indicator Survey, Demographic Health Survey, program data	[100–104]
Monitoring and assessment of service provision	Malaria, maternal health	1 (year) - 57 (month)	Facility, district	Kriging (ordinary kriging, space-time ordinary kriging, local space-time ordinary kriging); Bayesian geostatistical negative binomial model	Service Delivery Indicator Survey	[105–109]
Program evaluation	Malaria	36 (month)	District	Bayesian geostatistical models and Bayesian generalized linear models	Malaria Indicator Survey, malaria control program data, satellite data, Demographic Health Survey, ACTWatch household surveys	[110]
*Other cross-sectional analysis*						
Epidemiology	Maternal health	Median of 24 months	Province	Linear regression model	None	[111]
Monitoring and assessment of service provision	General, child health, maternal health, mental health	1 (year)	Facility, district, municipality, state	Descriptive statistics, Tobit regression model, bivariate and multivariate linear regression models,	Nutrition Service Delivery Assessment, abstraction from Integrated Nutrition Register, structured questionnaire with district health officers, District-level household and facility surveys, National Register of Health Service Providers, data from Institute of Geography and Statistics	[112–115]
Program evaluation	HIV and related diseases	1 (year)	District	Mixed-methods	Register reviews and a series of patient folder (health record) reviews	[51]

#### Time series analysis

Time series analysis using RHIS data was most often applied to evaluate programs and identify disease epidemiology, with one study assessing the impact of an infectious disease outbreak on primary health service utilization [82]. Studies analyzed indicators using large quantities of monthly or yearly data to estimate change (range of time units: 5–168). For instance, two-thirds of the studies analyzed three or more years of monthly data. Many of the studies utilized the highly disaggregated nature of the data by using either facility or district level data, with the exception of two studies which modelled national trends [33, 116]. Studies commonly applied strategies to account for temporal autocorrelation and the correlation between geographical units, including generalized linear models [58], multi-level analysis [77, 78], and ordinary least-squares regression with adjustment for seasonality and lag [34, 37, 117]. Among studies that modelled multiple facilities or administrative regions, random effects were commonly applied to account for heterogeneity.

In addition to RHIS data, a number of included studies incorporated data from external sources in their models based on geographical location such as district or region. Studies of malaria, for example, commonly included climate data from satellites in their models to control for important temporal factors, for example precipitation, humidity, and temperature [73, 117]. Other studies incorporated information from other national community surveys, health facility surveys, and program data as covariates [34, 77]. While most studies controlled for potential confounders by including covariates in analytic models, one study on maternal health service applied propensity score matching to further remove biases from differences in covariate distribution [37].

#### Geostatistical analysis

Geostatistical analyses using RHIS data were predominantly conducted for epidemiological purposes and the monitoring and assessment of service provision by exploiting geospatial information included in the RHIS at the facility or district level. Three of the studies that applied geostatistical analysis were cross-sectional, while the remainder were spatial-temporal. About half of the studies focused on malaria, of which three compared and illustrated various kriging methods to provide a reliable estimate of malaria burden amid missing reporting [105–107], and one study applied geostatistical modeling to select the most relevant health facility indicators for severe malaria outcomes [108]. Studies on other topics investigated the spatial or spatial-temporal dynamics of malaria in pregnancy [100], childhood diarrhea [101], clustering of malaria and HIV [102], and meningitis [118]. About half of the studies did not include data from external sources, and others triangulated data sourced from satellite data, Demographic and Health Surveys, national Malaria Indicator Surveys, and Service Delivery Indicator Surveys in their analyses. Studies that included covariates in the geostatistical analysis applied Bayesian hierarchical Poisson models or Bayesian geostatistical negative binomial models [103, 108, 110].

#### Pre-post comparison analysis

Pre-post comparison was commonly applied among studies that used RHIS data for program evaluation, and several studies used simple descriptive statistics to compare the periods before and after interventions. As pre-post comparison is subject to the limitation of temporal confounders and secular trends, two of the studies included contextual factors in regression modelling [35, 119].

#### Interrupted time series analysis

Most of the studies that conducted ITS analysis used it to evaluate interventions, and one assessed the impact of an infectious disease outbreak on maternal and child health service use [68]. The studies used large quantities of monthly data to model trend and level change (range of time unit: 44–132). RHIS data were minimally aggregated in these studies, which mostly analyzed facility or district level data, and similar to studies using time series analysis, accounted for autocorrelation through incorporating autoregressive structures or clustered standard errors in their modelling.

As ITS analyses are generally unaffected by confounding variables that do not change over time by design [120], baseline characteristics were typically not included in these models. Nonetheless, ITS analyses can be affected by time-varying confounders that rapidly change and some models included contextual factors from other data sources, such as climate and program data. To strengthen the quasi-experimental design, two studies also included a contrast group of time series to control for contextual changes that occurred at the same time as the interventions [38, 45].

#### Difference-in-difference analysis

Five studies applied difference-in-difference techniques using a wide range of time periods (range of time units: 4–48) and levels of geographical units (facility, district, provincial). Only one study included contextual characteristics from other data sources in its analysis. Analytic methods varied from descriptive comparison between and within intervention and control groups [41, 59, 87, 88], to ordinary least square regression with propensity score matching [42].

### Impact of research using RHIS data

**Fig. 3:**
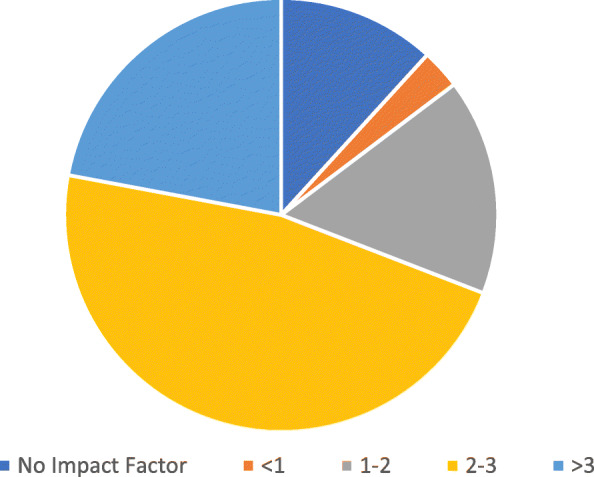
Distribution of impact factor of journals that published research studies that conducted statistical analysis of RHIS data (*n* = 68)

Most of the studies that conducted statistical analyses using RHIS data were published in journals with impact factors (88%, Fig. 3), two-thirds of which were two or higher, and more than a fifth of which were greater than three. Among those studies published in journals with the highest impact factors, most of them focused on program evaluation (53%), followed by monitoring and assessment of service provision (20%), epidemiology (20%) and impact evaluation (7%). These studies encompassed a range of health topics commonly studied using RHIS data.

### Strategies to circumvent RHIS data quality issues

Data quality is commonly cited as a barrier to using RHIS data in research, and slightly more than a quarter of the included studies described the strategies that they used to handle missing data and/or identify extreme values (Table 3). These strategies consisted of exclusion, imputation, interpolation, verification, and accounting for missing data in modeling. Exclusion of missing data was the most common practice, and among studies that used this technique, they excluded facilities from the analytic samples [38, 41, 45, 52, 65, 79, 83, 84, 87, 94, 96, 121], restricted the study period based on explicit criteria [54, 122], or applied sensitivity analysis to compare various exclusion criteria [41, 89, 90]. Imputation methods varied from assigning specific values to the missing observation [42, 87, 118, 123–125], to various modeling strategies such as conditional autoregressive model [110], generalized linear regression [124], and iterative singular value decomposition [124]. A sensitivity analysis was also conducted to select a specific imputation strategy [124]. Interpolation involves predicting values at unsampled locations. Methods described included the use of space-time kriging [105–107], and the adjustment of results by calibrating with other relevant information [52, 53, 55]. Some studies assumed data were missing at random, which was accounted for in specific modeling methods such as mixed-effect models [65, 124]. When the source of data could be reached, some studies also described verifying the missing information using registries where the original data were recorded [39, 73, 97, 111, 122].

**Table 3:** Strategies applied in research articles to counter issues of RHIS data

Type of strategy	Description of strategy
***Missing data***	
Exclusion	Exclude facility data if a certain threshold was reached (e.g. more than two-thirds of months in a year; more than a sixth of baseline data; facilities with any missing data)
	Restrict analysis to a period with a low level of missing data
	Sensitivity analysis to compare analysis of restricted period and full period
Imputation	Assign missing observations with mean-value for the year
	Assign missing observations with the average of precedent and subsequent data
	Imputation using conditional autoregressive model
	Missing value was replaced as positive (binary form) to prevent exaggeration of the fade-out effect
	Sensitivity analysis of imputation strategies: 1) single imputation using means, trimmed means, and median, 2) Poisson generalized linear modeling, 3) iterative singular value decomposition method
Interpolation	Interpolation using space-time kriging
	Adjust results by dividing each indicator by the percentage of reports submitted
	Adjust the data by calibrating to the total population using proportion reported in a household survey to have occurred in health facilities
Verification Account in the modeling method	Manual verification of the missing data with register at the health facility
	Missing data was assumed missing at random and accounted for in the mixed-effect models using standard maximum likelihood estimation
***Identifying extreme values***	
Specific threshold	Establishing a lower and upper limit based on proportion of the annual average or feasible value
	Univariate regression on individual facility-level to identify deviation from the mean time trend (e.g. if exceed 8 standard deviations)
Visual	Visual inspection of outliers
Analytic assessment	Jackknifing analysis to assess influence
	Student residual higher than an absolute value of 2 and influence on the estimated coefficients determined by high Cook’s distance statistics
***Handling of extreme values***	
Exclusion	Extreme values were excluded from analyses
Replacing extreme value with average	Extreme values were assigned the average value of the year; with exceptions of low average values
Replacing extreme value with missing	Outliers set to missing
Verification with data source	Any drastic change in monthly data reported electronically were manually verified with register at the health facility. Discrepancies were replaced with data in the register
Discount observation in estimation	Outliers were allocated a dummy coding to discount the observation in the calculation of coefficients
***Assess reliability***	
Data validation process	Randomly selected 10% of the total sample to check accuracy and reliability of data with reports and registers
	Verify data with another source (e.g. payroll)
	Established routine data validation process by health information and records officer (e.g. monthly data review meetings)

Slightly fewer articles described methods to identify and handle extreme values in the RHIS data, of which three types of strategies emerged: setting specific thresholds, visual inspection, and analytic assessment. Thresholds were set based on the distribution of the data, such as proportions or standard deviations from univariate regression. Several studies used visual inspection of outliers [38, 107], while the use of jackknifing analysis and the identification of influential points through Cook’s distance statistics were also applied [112, 126]. Upon identification of extreme values, several strategies were utilized: exclusion, replacement with the average value, replacement with the missing value, verification with a data source, or discounting the observation in statistical estimation. However, studies that replaced the extreme value with an explicit value potentially introduced bias into their estimates. A few studies also described the strategies applied to assess the reliability of the RHIS data, some of which were routine processes administered in the health systems [39, 97].

## Discussion

In recent years, there have been increased investments made to improve the quality of RHIS data in many LMICs. Over the same time period, we found an increase in published research using RHIS sourced data, especially over the past 5 years, likely due to the increased availability, accessibility, and quality of RHIS data [18]. While these studies have made contributions to the literature, we also found that the total number of studies conducted (*n* = 132) remains a small part of the overall literature base on health system evaluation and performance in LMICs.

Malaria and maternal health conditions were the most commonly studied health conditions, despite the fact that RHISs collect data on a wide range of other diseases and conditions. In particular, the use of RHIS data for non-communicable diseases (NCDs) research was very limited. As LMICs are undergoing an epidemiologic transition and the importance of NCDs is increasing [127], LMIC health systems face the increasing challenges of addressing the dual burden of communicable and non-communicable diseases [128, 129]. In spite of the limited implementation of non-communicable diseaseinterventions [129], the few studies that used RHIS data for non-communicable disease research mainly analyzed the gap in service provision and estimated disease burden, highlighting the large unmet need for health care in affected populations. A couple of the studies described how their research was limited by data availability and quality, such as the lack of diagnostic categories of the investigated health conditions in the RHIS. Future research should investigate how RHIS data on non-communicable diseases could better help to provide insights on its epidemiology and service provision to address these health conditions.

Our systematic review found that many of the studies took advantage of some of the features of RHIS data, in particular by exploiting the high frequency nature of these data at the level of health facilities, as well as combining external information to enhance estimations and enable assessing new research questions. The triangulation of populational health characteristics, environmental factors, and service coverage strengthens the analysis and the understanding of their influence [130]. In addition, the overlay of different information in analyses of RHIS data allows for the advancement of research methods. For instance, a recent study demonstrated how to assess the effects of facility readiness on severe malaria outcomes through constructing a composite facility readiness index based on health facility characteristics and spatial data, and using RHIS data as the outcome variable [108]. The detailed routine nature of RHIS data and the ability to link with other geographically based information, including data on population, environmental, health behavior, and facility characteristics, can generate high impact research and advance our understanding of disease epidemiology and health improvement efforts in LMICs.

Despite the increasing use of RHIS data for research purposes, the quality of these data remains imperfect and such issues should be identified and addressed in order to limit estimation error and bias. RHIS data quality issues remain a particular concern in some settings [131–133], however, other studies have shown that strategies that have been implemented to improve RHIS data across different international contexts can be successful [5, 134]. Multiple strategies were discussed in the articles we reviewed in our paper, including strategies to address common data quality issues such as missingness and data validity, for example the simple exclusion of missing data and various imputation and interpolation methods. However, the majority of the studies that used RHIS data did not describe the extent of the quality issues or the steps they took to overcome them. The use of sensitivity analyses in assessing the effect of specific cut-offs or methods was scarce. Explicit descriptions of the extent of the data quality issues and the reasons for selecting a particular approach should be encouraged in future research.

While our review used major databases and systematic methods, it nevertheless has some limitations that are worth noting. First, we included only peer-reviewed studies that were published in English, and therefore may have overlooked potentially relevant studies published in the grey literature or written in other languages. Additionally, given our focus on original research, we did not search the broader body of literature for books, reports, or grey literature. Our literature search also identified phrases that described health information systems in title and abstracts only, possibly resulting in the exclusion of studies that only mentioned RHIS data use in the full text. Finally, additional variants on these search terms may have generated more articles or a slightly different set of articles.

## Conclusions

In this systematic review we summarized the use of data collected from RHISs in LMICs. Overall, we found that researchers are increasingly using data sourced from RHISs to conduct health system planning and evaluation studies in LMIC health systems, however these data likely remain underutilized by the broader research community. As many of the studies included in this review were published in prominent journals and were able to use strong quasi-experimental or geo-spatial methods, we believe this makes the case for greater use of these data for research purposes in the future, which will likely happen as RHIS data become more openly available to the research community. However, there is a need to help build the case to use these data for a broader range of health conditions and to develop more of a consensus on methods to deal with data imperfections, given that our findings underlined the limited use and comparison of these methods. That said, our review clearly demonstrates the feasibility of use RHIS data in conjunction with rigorous study designs and analytic methods in LMICs. We suggest that future program evaluations should consider their use more broadly, to assess an increased variety of health conditions in conjunction with, or as a replacement for, household or facility survey methods.

## Supplementary information

**Additional file 1.**

## Data Availability

Not applicable.

## Abbreviations

DHIS 2: District health information system 2
HMIS: Health management information system
ITS: Interrupted time series
LMIC: Low- and middle-income country
NCD: Non-communicable disease
PRISMA: Preferred reporting items for systematic reviews and meta-analyses
RHIS: Routine health information system
