# Supplementary material for: Using routine health information data for research in low- and middle-income countries: a systematic review
Source: BMC Health Serv Res. 2020 Aug 25;20:790. doi: 10.1186/s12913-020-05660-1 (PMC7446185; doi:10.1186/s12913-020-05660-1)
Supplement: Supplementary file 1 — Additional file 1. [file 12913_2020_5660_MOESM1_ESM.docx]

**Appendix 1**

| CONCEPT | KEYWORDS |
| --- | --- |
| Routine Health Information System | "routine health information system*" OR "routine health management information system*" OR "health management information system*" OR "health information system*" |
| Low- or Middle-Income Country | "Albania"  OR  "Algeria"  OR  "American Samoa"  OR  "Angola"  OR  "Armenia"  OR  "Azerbaijan"  OR  "Bangladesh"  OR  "Belarus"  OR  "Belize"  OR  "Benin"  OR  "Bhutan"  OR  "Bolivia"  OR  "Bosnia and Herzegovina"  OR  "Botswana"  OR  "Brazil"  OR  "Bulgaria"  OR  "Burkina Faso"  OR  "Burundi"  OR  "Cabo Verde"  OR  "Cambodia"  OR  "Cameroon"  OR  "Central African Republic"  OR  "Chad"  OR  "China"  OR  "Colombia"  OR  "Comoros"  OR  "Democratic Republic of Congo"  OR  "Republic of Congo"  OR  "Costa Rica"  OR  "Cote d'Ivoire"  OR  "Cuba"  OR  "Djibouti"  OR  "Dominica"  OR  "Dominican Republic"  OR  "Ecuador"  OR  "Egypt"  OR  "El Salvador"  OR  "Equatorial Guinea"  OR  "Eritrea"  OR  "Ethiopia"  OR  "Fiji"  OR  "Gabon"  OR  "Gambia"  OR  "Georgia"  OR  "Ghana"  OR  "Grenada"  OR  "Guatemala"  OR  "Guinea"  OR  "Guinea-Bissau"  OR  "Guyana"  OR  "Haiti"  OR  "Honduras"  OR  "India"  OR  "Indonesia"  OR  "Iran"  OR  "Iraq"  OR  "Jamaica"  OR  "Jordan"  OR  "Kazakhstan"  OR  "Kenya"  OR  "Kiribati"  OR  "North Korea"  OR  "Kosovo"  OR  "Kyrgyz Republic"  OR  "Lao PDR"  OR  "Lebanon"  OR  "Lesotho"  OR  "Liberia"  OR  "Libya"  OR  "Macedonia"  OR  "Madagascar"  OR  "Malawi"  OR  "Malaysia"  OR  "Maldives"  OR  "Mali"  OR  "Marshall Islands"  OR  "Mauritania"  OR  "Mauritius"  OR  "Mexico"  OR  "Micronesia"  OR  "Moldova"  OR  "Mongolia"  OR  "Montenegro"  OR  "Morocco"  OR  "Mozambique"  OR  "Myanmar"  OR  "Namibia"  OR  "Nauru"  OR  "Nepal"  OR  "Nicaragua"  OR  "Niger"  OR  "Nigeria"  OR  "Pakistan"  OR  "Papua New Guinea"  OR  "Paraguay"  OR  "Peru"  OR  "Philippines"  OR  "Romania"  OR  "Russian Federation"  OR  "Rwanda"  OR  "Samoa"  OR  "Sao Tome and Principe"  OR  "Senegal"  OR  "Serbia"  OR  "Sierra Leone"  OR  "Solomon Islands"  OR  "Somalia"  OR  "South Africa"  OR  "South Sudan"  OR  "Sri Lanka"  OR  "St. Lucia"  OR  "St. Vincent and the Grenadines"  OR  "Sudan"  OR  "Suriname"  OR  "Swaziland"  OR  "Syrian Arab Republic"  OR  "Tajikistan"  OR  "Tanzania"  OR  "Thailand"  OR  "Timor-Leste"  OR  "Togo"  OR  "Tonga"  OR  "Tunisia"  OR  "Turkey"  OR  "Turkmenistan"  OR  "Tuvalu"  OR  "Uganda"  OR  "Ukraine"  OR  "Uzbekistan"  OR  "Vanuatu"  OR  "Venezuela"  OR  "Vietnam"  OR  "West Bank and Gaza"  OR  "Yemen"  OR  "Zambia"  OR  "Zimbabwe" |

Appendix Table 1. Database search strategy
